# Flying enhances viewing from above bias on ambiguous visual stimuli

**DOI:** 10.1167/jov.23.6.11

**Published:** 2023-06-19

**Authors:** Xue Zhang, Qilong Tan, Haiying Mu

**Affiliations:** 1 Institute of Aviation Human Factors and Cognitive Neuroscience, Department of Aviation Psychology, Flight Technology College, Civil Aviation Flight University of China, Guanghan, China; 2 Institute of Aviation Human Factors and Cognitive Neuroscience, Department of Aviation Psychology, Flight Technology College, Civil Aviation Flight University of China, Guanghan, China; 3 Institute of Aviation Human Factors and Cognitive Neuroscience, Department of Aviation Psychology, Flight Technology College, Civil Aviation Flight University of China, Guanghan, China

**Keywords:** ambiguous vision, spatial disorientation, point-light walker, viewing from above bias

## Abstract

The human spatial orientation system is well designed on the ground but is imperfect in the aeronautical three-dimensional (3D) environment. However, human perception systems perform Bayesian statistics based on encountered environments and form shortcuts to improve perceptual efficiency. It is unknown whether our perception of spatial orientation is modified by flying experience and forms perceptual biases. The current study tested pilot perceptual biases on ambiguous visual stimuli, the bistable point-light walkers, and found that flying experiences increased the pilot's tendency to perceive himself as higher than the target and the target as farther away from them. Such perceptual effects due to flight are likely to be attributed to experience of variable vestibular state in a higher position in 3D space, rather than the experience of a higher viewpoint. Our findings suggest that flying experience will modifies our visual perceptual biases, and that more attention should be paid to the enhanced viewing from above bias when flying to avoid overestimating altitude or viewing angle when the visual conditions are ambiguous.

## Introduction

Over tens of millions of years of mammalian evolution, we humans have developed a well-designed system of spatial orientation in two dimensions. However, this system becomes imperfect when we enter the aeronautical three-dimensional (3D) environment (Previc & Ercoline; 2004). In the evolutionary history of life, the once-ground-dwelling theropods evolved feathers and wings, dramatically reduced their body size, and transformed to birds (Lee, Cau, Naish, & Dyke, 2014; Xu et al., 2014). Analogously, to adapt to a resource-constrained environment, we humans have evolved larger brains (Chaline, 2003), through which we have created aircraft and expanded our activities into the sky since the early 20th century. From the first moment, however, when people began to soar over the heavens, our system of spatial orientation was confronted with the problem of adaptation to a new environment (Henn & Young, 1975; Previc & Ercoline, 2004). For instance, sudden acceleration or deceleration during level flight can confuse the vestibular system into misinterpreting the motion as a climb or dive, because of the altered gravitoinertial force in 3D space combined with ambiguous visual cues (Previc, Varner, & Gillingham, 1992; Tokumaru, Kaida, Ashida, Mizumoto, & Tatsuno, 1998). Similar misperceptions of position, altitude, or motion relative to the ground plane are termed as *spatial disorientation* (Kirkham et al., 1978).

Besides the limitation of evolution, human spatial orientation system is also driven by the Bayesian statistics on encountered environment, as captured by “prior probability” distributions (Cheng, Shettleworth, Huttenlocher, & Rieser, 2007; Haselton, Nettle, & Murray, 2015; MacNeilage, Banks, Berger, & Bulthoff, 2007). Such priors induce perceptual biases toward one interpretation over other alternatives. In aviation, ambiguous spatial visual cues will be interpreted as cues for preferred orientation in an emergency. Here we ask whether our perception of spatial orientation would be modified by flying experience and forms a particular perceptual bias. Existing literature shows that humans have a rich experience of light source coming from above, and therefore pilots are prone to experience inversion illusions when the ground is brighter than the sky (Mamassian & Goutcher, 2001; Vinacke, 1948). In addition, flying activity itself will inevitably modify the prior probability of pilot self-orientation, such as the probability of higher spatial locations with more complex vestibular senses and higher viewpoints with ambiguous visual conditions. In perception, flying experience increases the pilot's ability to perceive pitch angular displacement through the adaptation of the vestibular system (Tribukait & Eiken, 2012). In cognition, flying experience can help pilots with verbal cognitive styles to reduce the degree of confirmation bias, a tendency to seek out and interpret information in a way that confirms preexisting beliefs, expectations, or hypotheses during decision making (Xu et al., 2022). Thus we expect that these novel flying experiences will also modify the pilot's perceptual inferences about spatial orientations in the presence of ambiguous visual stimuli.

**Figure 1.**
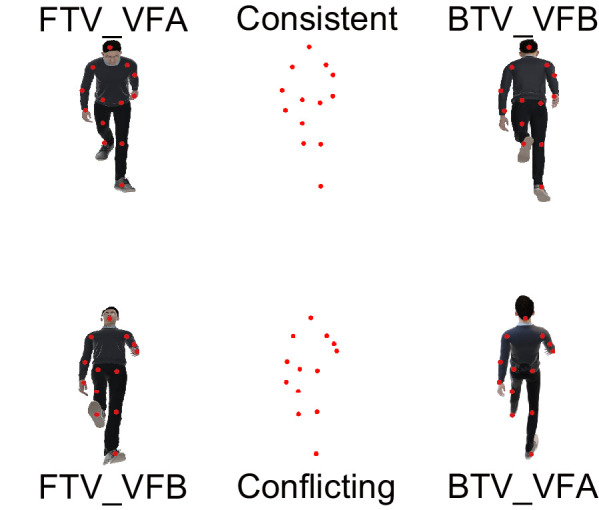
Illustrations of the visual stimuli in the experiments. The dots in the middle illustrate two different bistable PLW stimuli (consistent: FTV and VFA percepts are combined in a plausible interpretation; conflicting: either FTV bias or VFA bias dominate). The red color of the dots is only used here for illustration. The adjacent mannequins attached with red dots identical to those in the middle display the corresponding possible percepts of the bistable stimuli (FTV_VFA, facing toward the viewer and viewing from above; FBV_VFB, facing backward the viewer and viewing from below; FTV_VFB, facing toward the viewer and viewing from below; FBV_VFA, facing backward the viewer and viewing from above).

To measure changes in such perceptual inference, the current study adopted ambiguous visual stimuli, which have two plausible interpretations. Specifically, as shown in Figure 1, the stimuli are point-light walkers (PLWs), which are ambiguous in both horizontal directions and vertical viewpoints, thus leading to a bias of facing toward the viewer (FTV) and a bias of viewing from above (VFA) (Vanrie & Verfaillie, 2004; Zhang, Xu, Jiang, & Wang, 2017). The VFA bias is in the dimension of the vertical direction, while the FTV bias is in the dimension of the target face direction. Some minor perceptual bias of spatial orientation in these two dimensions may cause spatial disorientation in pilots (Gibb, 2007). In the current study, we first measured the perceptual interpretations of bistable PLWs on pilot cadets after flying and on pilot cadets during a two-day break on the ground. If flying could modify pilots’ perceptual inferences when visual cues are ambiguous, the rate of perceptual biases would be significantly different between the two groups. Then, to further explore which novel components of the flying experience influence spatial orientation perceptual biases, we tested the perceptual interpretations of bistable PLWs on expert pilots resting on the ground and pilot cadets after simulated flights.

## Methods

### Participants

In total, four groups of participants (male pilots with a mean age of 34 or male pilot cadets with a mean age of 20) with different flight experiences were included in the current study. All these participants were recruited during their flight or theoretical training courses in 2019 to 2020. The post-flight group was formed by 21 randomly recruited pilot cadets who were undergoing flight training at Guanghan Airport of Civil Aviation Flight University of China. They were required to complete the test immediately after they got off the plane after a flight of at least one hour. The ground-resting group consisted of 21 randomly recruited pilot cadets who were taking a two-day break from flight training. The post-simulated flight group was formed by 16 randomly recruited pilot cadets who were undergoing simulated flight training. They were required to complete the test immediately after completing approximately one hour of simulated private pilot flying in the Microsoft Flight Simulator X. The expert group during the break consisted of 15 airline pilots with an average of 9433 hours of flying experience. They were recruited during a 2-month theoretical training course. They had not flown for at least two weeks. Participants with extremely low correct rate data were excluded from further analyses (see below for data analyses, three for post-flight group, four for ground-resting group, three for post-simulated flight group, two for ground-resting experts group). All participants had normal or corrected-to-normal vision and were naive to the purpose of the experiment. They gave written informed consent, which was approved by the institutional review board of the Civil Aviation Flight University of China, and received course credits.

### Stimuli

Stimuli are white PLW on a gray background, generated and displayed using MATLAB together with the Psychophysics Toolbox extensions (Brainard, 1997; Pelli, 1997). As shown in Figure 1, the point-light consists of 13 white dots placed at the major joints and the head of a human walker. The walking motion is formed by 30 frames of 3D animation of a real person walking on a treadmill. The 3D animation was then projected orthographically onto the screen, resulting in a lack of depth cues. In this way, the PLW can be perceived as either facing towards or backwards the viewer (Vanrie & Verfaillie, 2004; Zhang et al., 2017). Typically, it is perceived as facing toward the viewer, which is known as FTV bias. To produce the VFA bias, we rotate this bistable walker by some degree with respect to the horizontal plane to change the vertical angle of deviation. Similarly, the vertical angle of deviation can be considered as either the elevation angle or the depression angle, resulting in either viewing from above or viewing from below. By this means, we generate two types of ambiguous PLW stimuli (consistent: FTV and VFA percepts are combined in a plausible interpretation; conflicting: either FTV bias or the VFA bias dominate). Compared to bistable stimuli with only one ambiguous parameter, these PLWs are advantageous in avoiding random responses from participants. Because only two out four pairs of responses (Figure 1) are meaningful for each bistable stimulus with ambiguities in two dimensions. In the present study, bistable stimuli, which cause two biases to conflict, were used as an optimal tool to measure subtle changes in perceptual biases. Meanwhile, stimuli with consistent perceptual biases were used to check for the presence of perceptual biases. Moreover, we also wonder to what extent the experience of flying modulates perceptual bias. Thus we set five vertical angles with gradients varying from 25° to 5°.

### Procedure

Participants sat in front of a 22-inch liquid crystal display (1280 × 1024, 60 Hz) with a viewing distance of 60 cm. Each trial began with a PLW stimulus displayed on a gray background with a white fixation cross in the center of the screen. The stimuli (ranging from 5.7° to 6.7° in height, depending on the angular deviation from the horizontal plane) were presented in 0° from the sagittal plane. Before the formal test, we displayed a 360° rotatable PLW in three dimensions to ensure that the observer understood the different viewpoints of the PLW. Note here that the observers should not be informed that the PLW was bistable. The observers were then subjected to practice trials to familiarize themselves with the procedure. The PLW stimulus was displayed for 1000 ms in each trial. The observers were then asked to judge (1) the walking direction of the PLW (e.g., toward the viewer or backward from the viewer) and (2) the viewpoint with respect to the horizontal plane (viewing from above or from below) by successively pressing the corresponding keys. There were 100 trials in total, 10 trials for each of the 10 stimuli (consistent and conflicting ones combined with five vertical angles). All trials were run in random order. All tests were performed in a quiet separate room to avoid interference from irrelevant factors.

### Data analyses

The analyses included only the correct trials whose pairwise response matched one of the two plausible interpretations for the ambiguous stimulus. For example, in Figure 1, it is false if the pairwise response is FTV_VFB for the upper PLW stimulus. At each vertical angle, the rate for each response type was computed by dividing the number of trials with that response type by the number of correct trials. For conflicting stimuli, to quantify bias competition, an index of bias competition was computed by subtracting the rate of FTV using the rate of VFA. We excluded participants whose correct rate was zero for at least one angle, in which case, it was impossible to compute a rate.

## Results

First, we probed the effect of fresh experience of flying on the observer's perceptual biases. We randomly recruited a number of pilot cadets who were in flight training. Half of them belonged to an experimental group, which was required to fly for at least one hour in the sky before observing ambiguous PLWs. The other half was a control group, in which participants were given at least two days of rest before observing the same stimuli used in the experimental group.

**Figure 2.**
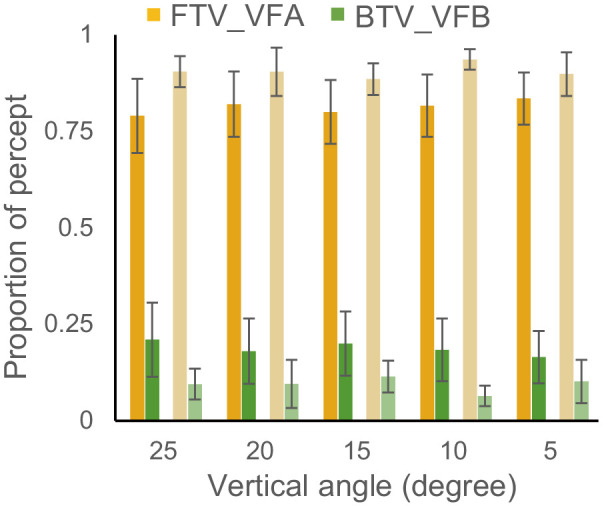
Mean proportions of the two possible responses to consistent stimuli. Conditions were five vertical angles (25°, 20°, 15°, 10°, 5°) × 2 groups of participants with different flying experiences (Dark color: pilot cadets after flying; light color: pilot cadets during a two-day break on the ground after flight training). Error bars denote ±SEM.

We collected the responses of the observers on the walking directions of the PLWs and the corresponding viewpoints. Trials with incorrect responses and data from subjects with low correct rates (see methods) were excluded from the statistics. If observers had pronounced perceptual biases, their responses to consistent stimuli would be biased toward the preferred interpretation of ambiguous stimuli. But for conflicting stimuli, the two biases would counter each other and were therefore hard to detect. To confirm the existence of perceptual biases, we should first look at the proportions of two types of responses for consistent stimuli. The mean proportions and standard error mean of all five angles and two groups are shown in Figure 2. Within each group, we compared the proportions of the two types of responses across all five angles using repeated measures analysis of variance (ANOVA). As shown in Figure 2, observers from both groups preferred the FTV_VFA perceptually with striking significance (group after flying: *F*(1, 17) = 15.97, *p* < 0.001, $\eta_{p}^{2}$ = 0.48; group rest on ground: *F*(1, 16) = 126.13, *p* < 0.001, $\eta_{p}^{2}$ = 0.89). This was in agreement with previous study showing the cooperation of FTV and VFA biases (Zhang et al., 2017). So, whether such cooperation was different between groups? Next, we used a mixed-design ANOVA to compare the dominant FTV_VFA percept between the two groups and across vertical angles. The results showed no significant difference between the groups (*F*(1, 33) = 1.13, *p* = 0.30, $\eta_{p}^{2}$ = 0.03 ) and across vertical angles (*F*(4, 132) = 0.36, *p* = 0.83, $\eta_{p}^{2}$ = 0.01). So, there were indeed perceptual biases in interpreting these ambiguous visual stimuli, but it is hard to observe any effect of flying experience on perceptual biases for consistent stimuli.

**Figure 3.**
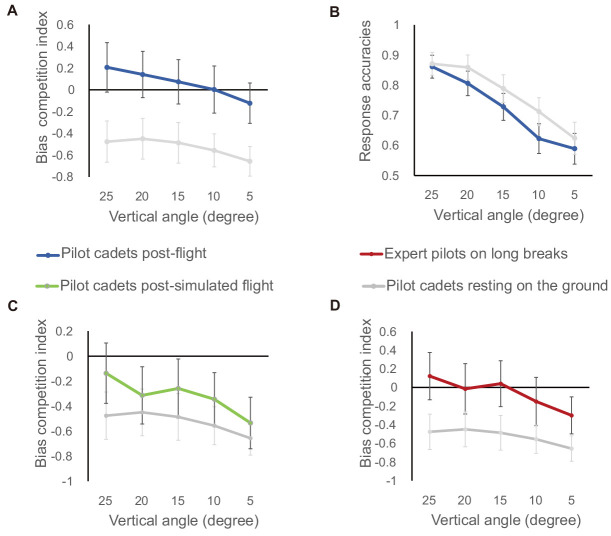
Results for all four groups of participants. (A, C, D)The competition index of two perceptual biases (the FTV bias and the VFA bias) for the conflicting stimuli as a function of vertical angular deviation (25°, 20°, 15°, 10°, 5°). (B) The response correct rates for the group of pilot cadets after flying and for the group of pilot cadets during a two-day ground rest period after flight training. Error bars denote ± SEM.

However, perception will be systematically biased under conditions where utility and accuracy conflict with one another (Martin, Solms, & Sterzer, 2021). Previous studies had categorized FTV bias as a type of error management bias and VFA bias as a type of statistics-based bias (Johnson, Blumstein, Fowler, & Haselton, 2013; Troje & McAdam, 2010; Zhang et al., 2017). For conflicting stimuli, the competing of two biases would fluctuate the rate of FTV and VFA percepts. As a result, only one bias could dominate the observer's perception (Zhang et al., 2017). From this, we computed a biases competition index (rate_VFA_ − rate_FTV_) to quantify biases competition. Here, a larger index corresponds to a stronger VFA bias or a weaker FTV bias. To test whether flying could modulate pilot perceptual biases, we compared the bias competition index across vertical angles between the experimental and control groups using a mixed-design ANOVA. As shown in Figure 3A, the bias index decreased significantly with decreasing vertical angle (*F*(4, 132) = 3.51, *p* < 0.01, $\eta_{p}^{2}$ = 0.10) and was significantly larger after the flight than after two days of ground rest (*F*(1, 33) = 5.30, *p* < 0.05, $\eta_{p}^{2}$ = 0.14). And there was no significant interaction between the vertical angle and the group (*F*(4, 132) = 0.28, *p* = 0.89, $\eta_{p}^{2}$ = 0.01). In other words, the strength of the VFA bias dropped whereas the strength of the FTV bias rose as the vertical angle decreased. This result was consistent with our previous study (Zhang et al., 2017). Meanwhile, a more important result was that the VFA bias rose significantly after flight. A post hoc comparison of bias indexes between the two groups showed that such an enhancement of the VFA bias was observed not only at larger but also at smaller vertical viewpoints (Table 1, 25°: *t*(33) = 2.29, *p* < 0.05, *d* = 0.73; 20°: *t*(33) = 2.08, *p* < 0.05, *d* = 0.67; 15°: *t*(33) = 2.02, *p* < 0.05, *d* = 0.65; 10°: *t*(33) = 2.09, *p* < 0.05, *d* = 0.68; 5°: *t*(33) = 2.29, *p* < 0.05, *d* = 0.73). This result suggests that flight activity may increase the tendency of pilots to perceive themselves as higher than the target, which is moving away when the visual conditions are ambiguous.

**Table 1.** Strengthened biases competition index for different groups relative to the control group (N = 17). *Notes:* ^*^ *p* < 0.05; ^†^*p* < 0.1.

Groups	No.	25°	20°	15°	10°	5°
Pilot cadets post-flight	18	0.69^*^	0.59^*^	0.56^†^	0.56^*^	0.54^*^
Pilot cadets post-simulated flight	13	0.34	0.14	0.23	0.21	0.12
Expert pilots on long breaks	13	0.6^†^	0.43	0.53^†^	0.41	0.36

One might argue that flying would change the pilot's perceptual sensitivities on judging their viewpoints and targets’ moving directions. Existing literature reports that flight experience increases the sensitivity to perceive pitch angular displacements (Tribukait & Eiken, 2012). Thus it is necessary to rule out this possibility. Here, we used the mixed-design ANVOA to compare the correct rate at all five angles for conflicting stimuli between the two groups. The results are shown in Figure 3B, where the correct rate decreased significantly as the vertical angle decreased (*F*(4, 132) = 19.22, *p* < 0.001, $\eta_{p}^{2}$ = 0.37). But there was no significant difference between the two groups (*F*(1, 33) = 1.16, *p* = 0.29, $\eta_{p}^{2}$ = 0.03). It is therefore unlikely that pilots’ sensitivities play a key role in modulating the intensity of perceptual bias after flight.

So far, we have observed that flying definitely modifies our perceptual biases. Existing literature proposed that the perceptual bias could be modulated by prior knowledge about the environment (Harrison & Backus, 2010). In our study, flying undoubtedly changed our brain's Bayesian statistics of the environment. However, the vestibular state and visual experience of pilots on the sky were simultaneously altered. So, we further asked which factors in the flight mainly contributed to the bias shift observed above. We then recruited another group of pilot cadets, who were training in simulated flight on ground. They had just gone through an hour-long visual experience similar to pilot cadets flying in the sky before observing the ambiguous PLW, yet they wouldn't experience a variable vestibular state at a higher spatial position than most targets. We compared the bias index between this group and the control group at all vertical angles using a mixed-design ANOVA. If visual experiences played important role, we would get a bias shift effect here. In contrast, the results shown in Figure 3C and Table 1 show that they had no significant bias shift from the control group (*F*(1, 28) = 0.64, *p* = 0.43, $\eta_{p}^{2}$ = 0.02). This suggests that the visual experience at higher viewpoints is insufficient to produce a significant shift in the VFA and FTV bias.

Because visual experience of higher viewpoints had no effect, we instead tested whether more experience of variable vestibular states during flight could make the VFA bias stronger. We recruited a group of expert pilots with mean flight experience of 9433 hours and at least two weeks on the ground. They definitely had more experience of flying at higher altitudes in space than the average person. They were asked to observe PLWs following a procedure identical to that followed by the group of pilot cadets resting on the ground. Their bias index, displayed in Figure 3D, appears larger than the control group's at all angles and was marginally significant (*F*(1, 28) = 2.84, *p* = 0.10, $\eta_{p}^{2}$ = 0.09). Specifically, as shown in Table 1, a post hoc comparison of the bias index between expert pilots and pilot cadets during ground rest revealed that the expert pilots had a larger bias index at angles of 25° (*t*(28) = 1.93, *p* < 0.10, *d* = 0.68) and 15° (*t*(28) = 1.74, *p* < 0.10, *d* = 0.62). This result suggests that the factor of experiencing more variable vestibular states at higher spatial positions than most targets plays a part role in the perceptual bias shift.

## Discussion

The current study tested the pilot cadets’ perceptual interpretations of bistable PLWs after flight. We found that they increased their tendency to perceive themselves as higher than PLWs who were walking away from them. The effect could not be attributed to a variation in pilot's sensitivity after flight. In addition, expert pilots even showed minor effects on the ground, but pilot cadets experienced not a significant perceptual bias shift after a simulated flight on the ground. We thus infer that the experience of variable vestibular states during flight at higher spatial positions than most targets, rather than the experience of higher viewpoints, plays a key role. Such a perceptual tendency becomes analogous to a bird of prey's preferred judgement of the orientation of its target. It is a perceptual shortcut that helps aviators to perceive pitch angles and altitudes more efficiently in emergency or ambiguous situations. However, if the reality is opposite to the result obtained from the shortcut, such enhanced perceptual bias can lead to spatial disorientation and pose a potential risk to human aviation.

Our findings that immediate experience of flying or more experience of flying can strengthen perceptual bias are actually common in visual perception. Researchers in the field of vision have proposed that an efficient visual system should change its approach to resolving ambiguities in two-dimensional information after being exposed to new experiences. Actually, we can establish more efficient perceptual biases in the natural world after more experiences of resolving ambiguous visual stimuli that are not easily interpreted by existing visual rules (Harrison & Backus, 2010). The same rule applies to spatial orientation perception in aviation. Pilots often experience somatogravic illusion, an erroneous “nose-up” pitch perception that occurs when the plane accelerates forward, because of the pilot's misinterpretation of gravitoinertial force as gravity (Carriot, Barraud, Nougier, & Cian, 2006; Previc et al., 1992). A Bayesian model for the disambiguation of gravitoinertial force by adding visual cues indicating orientation and acceleration with respect to the Earth verified that the accuracy of spatial orientation perception can be improved by adding a prior of visual cues into the Bayesian model (MacNeilage et al., 2007). In neural sciences, researchers have proposed that our brains optimize performance through experience. It has been proposed that perceptual repetition facilitates neural activities within and between the percept-specific visual networks and the parietal networks involved in temporal integration of perceptual information. Such neural activities lead us to form the biases on previously experienced percepts (de Jong, Kourtzi, & van Ee, 2012). From this, we infer that enhanced perceptual bias after experiencing flight in 3D space is an optimization of the human visual system that can help humans more effectively resolve ambiguous visual information in future flights.

Although the stronger perceptual bias increases the pilot's visual perception efficiency, it also increases the probability of perceiving inaccurate or even opposite spatial orientation during emergency troubleshooting or in ambiguous weather conditions. If the pilot has a stronger VFA bias, the viewing angle between themselves and other aircraft or obstacles may be overestimated. Even when they are below the target, they may mistakenly assume that they are above the obstacle. It is well known that a false estimate of the viewing angle can sometimes be fatal to a pilot. For example, fighter-pilots during night training wearing night-vision goggles are prone to improperly estimate the elevation of their target while accomplishing a high-altitude strafe, resulting in impact on the ground (Gibb, Ercoline, & Scharff, 2011). According to the Aviation Safety office release of aeromedical causes of mishaps from 1990 to 2008, the first cause was spatial disorientation, followed by “visual illusions” at no. 4 (Gibb et al., 2011). For spatial orientation perception, both visual and vestibular perception contribute interactively, with vision accounting for nearly 80% in the aerospace environments (Sanchez-Tena, Alvarez-Peregrina, Valbuena-Iglesias, & Palomera, 2018). Therefore our findings on the visual perceptual bias shift of visual perception in the current study should be given more attention in future flights with poor visual conditions to avoid the type of unrecognized spatial disorientation that is always a fatal issue for the safety of pilots (Hao et al., 2020).

The flight can also change the pilot's vestibular state. During the flight, pilots will go through both ascending and descending states, under which they may frequently experience the feelings of being overweight and weightless. Neuroscientists have demonstrated the existence of such a vestibular cortex in the posterior insula region, which represents the visual gravitational motion (Indovina et al., 2005). In addition, there is an interaction between the visual and vestibular systems (Brandt, Bartenstein, Janek, & Dieterich, 1998). Such interaction is more related to spatial cognitive processing (Furman, Redfern, Fuhrman, & Jennings, 2012). In our study, the post-flight vestibular systems of pilot cadets had just received several hours of unstable stimulation, which might have affected their perceptual bias toward vertical direction through visual-gravitational interaction. However, expert pilots, after thousands of hours of unstable vestibular stimulation, might have reconstructed their cognitive and neural models of visual gravitational motion to optimize visual processing in vertical directions. Consistent with our inference, a study has demonstrated that vestibular cortex lesions affect the perception of verticality (Brandt, Dieterich, & Danek, 1994). In addition, our previous study found that prolonged microgravity reduced the inversion effect of perception on point-light walker orientation by enhancing the visuo-vestibular interaction between cortical regions dedicated to visual biological motion processing and vestibular gravity estimation (Wang et al., 2022). To demonstrate our speculation, future research should focus on changes in functional connectivity between brain regions dedicated to visual-vestibular interactions after flight.

We also noticed that our visual system often perceives the opposite interpretation of a bistable stimulus after a period of perceiving the dominant interpretation (Leopold & Logothetis, 1999; Sterzer, Kleinschmidt, & Rees, 2009). For instance, after observing a PLW with an explicit heading direction, the observer experiences an aftereffect when viewing a bistable PLW that is perceived reversed in depth (Jackson & Blake, 2010). The researchers suggested that neurons associated with the dominant percept were adapted to reduce their sensitivity, so that the observer was more likely to perceive the opposite in the following test (Lankheet, 2006; Toppino & Long, 2015). Nevertheless, in this study, we did not observe any such phenomenon. First, it has been suggested that both adaptation and noise affect the rivalry of the two percepts (Moreno-Bote, Rinzel, & Rubin, 2007; Shpiro, Moreno-Bote, Rubin, & Rinzel, 2009). Moreover, adaptation of neural activities after uninterrupted prolonged observation of ambiguous visual stimuli usually changes the dominant perception. In our study, observers had experienced variable vestibular states at higher positions in 3D space, and their visual system was not continuously fixed at lower ground. This might introduce noises to interrupt the adaption of neurons associated with viewing from above the target. This inference is in agreement with the proposal of a review of ambiguous vision that perception tends to retain the original dominant interpretation when ambiguous stimuli are presented briefly with intervening blank periods due to sensory memory (Pearson & Brascamp, 2008). Actually, study of pilot visual patterns has shown that pilots’ fixations change frequently between the Head Up Display (HUD) and the outside of the cockpit (Yu et al., 2016). For the above reasons, the effect of neural adaptation might be too weak relative to the effect of Bayesian statistics of fresh experiences to affect the following perceptual biases in our study.

## Conclusions

In summary, expert pilots or pilot cadets after flying had enhanced their tendency to perceive themselves as higher than the ambiguous targets. Experiences at higher spatial positions associated with fluctuating vestibular states played an important role. Pilot visual gravitational model at vestibular cortex and Bayesian statistics of self-orientation might be modified to raise their visual cognition efficiency. But here we remind pilots to pay more attention to viewing from above bias to avoid overestimating their altitude before landing or viewing angles between themselves and other aircraft or obstacles in ambiguous visual conditions. Therefore to reduce pilot human error and improve aviation safety, pilots should undergo specific training in vertical viewpoint perception after vestibular training to overcome the enhanced VFA bias. However, our findings came from pilots or pilot cadets during training who were not alerted to overestimating altitude. In future studies, to generalize our findings, we should also test for these perceptual biases in pilots on duty and explore whether the effect disappears after pilots are alerted.
